# Live performance, nutrient digestibility, immune response and fecal microbial load modulation in Japanese quails fed a *Bacillus*-based probiotic alone or combination with xylanase

**DOI:** 10.1080/01652176.2024.2364641

**Published:** 2024-06-21

**Authors:** Asad Sultan, Syed Murtaza, Shabana Naz, Ziaul Islam, Abdulwahed Fahad Alrefaei, Rifat Ullah Khan, Samia H. Abdelrahman, A. Chandrasekaran

**Affiliations:** a Department of Poultry Science, Faculty of Animal Husbandry and Veterinary Sciences, The University of Agriculture, Peshawar, Pakistan; b Department of Zoology, Government College University, Faisalabad, Pakistan; c Department of Animal Science, Shaheed Benazir Bhutto University Sheringal Dir Upper, Sheringal, Pakistan; d Department of Zoology, College of Sciences, King Saud University, Riyadh, Saudi Arabia; e College of Veterinary Sciences, Faculty of Animal Husbandry and Veterinary Sciences, The University of Agriculture, Peshawar, Pakistan; f Central Laboratory, Khartum, Sudan; g Department of Physics, Sri Sivasubramaniya Nadar College of Engineering, Kalavakkam, Tamil Nadu, India

**Keywords:** Beneficial bacteria, enzyme supplementation, feed additive, growth promotors, health

## Abstract

Animal industry seeks cost-effective solutions to enhance performance and health of domestic animals. This study investigated the effects of supplementing *Bacillus* spp. probiotics and xylanase on 2000 one-day-old Japanese quails, randomly assigned to four treatment groups (10 replicates). The control group received no supplementation, while the others were supplemented with a *Bacillus*-based probiotic at 7.5 × 10^7^ cfu/kg of feed, xylanase enzyme (2,000 U/kg) alone or in combination. Quails receiving both probiotic and enzyme exhibited significantly (*p* < 0.01) higher weekly and overall weight gain, and lower feed conversion ratios compared to the control group. Dressing percentage was higher (*p* < 0.01), and mortality lower in birds supplemented with a combination of enzyme and probiotic. Antibody titres against infectious bronchitis and infectious bursal disease were significantly (*p* < 0.01) higher in quails receiving combined probiotic and enzyme supplementation, while titres against Newcastle disease virus were higher (*p* < 0.01) in groups supplemented with probiotic and enzyme individually or in combination. Additionally, digestibility was significantly (*p* < 0.01) higher in groups receiving combined enzyme and probiotic supplementation, with higher apparent metabolizable energy compared to the control. The populations of beneficial *Lactobacillus* increased, while harmful *E. coli* and *Salmonella* decreased significantly in quails supplemented with both probiotic and enzyme. In conclusion, supplementing xylanase enzyme and probiotic together in Japanese quails positively influenced growth, nutrient digestibility, immune response, and cecal microbiota.

## Introduction

Feed supplementsare pivotal in the animal production and health, particularly in the production of human-consumable protein, with a primary focus on monogastric animals (Landy et al. 2021; Hegazy et al. 2022; Sultanayeva et al. 2022; Imtiaz et al. 2023; Hafeez et al. 2024). In 2006, the use of antibiotics as antimicrobial growth promoters was prohibited in poultry feed due to concerns regarding antibiotic residues in meat and eggs, impacting poultry production and health. Monogastric animals, particularly poultry, often lack the ability to produce sufficient endogenous enzymes for breaking down nutrients and antinutritional factors in their feed (Ravindran and Abdollahi 2021). Traditionally, exogenous enzymes have been employed as feed supplment to enhance nutrients digestability. However, there is a recent trend in using feed additives that go beyond enzymes, such as probiotics. These microbes are anticipated to bolster the immune system through various mechanisms, offering viable alternatives to the use of antimicrobials as growth promoters (Nusairat et al. 2022).

Feed enzymes, designed to enhance nutrient digestibility, have been shown to improve overall animal performance and facilitate the incorporation of more economical feed materials , providing formulation flexibility (Flores et al. 2016; Hafeez et al. 2020; Elihasridas et al. 2023). Moreover, effective combinations of exogenous enzymes and substrates allow for the utilization of a many non-conventional byproduct ingredients (Jabbar et al. 2021), reducing feed costs and promoting the recycling of byproducts, contributing to sustainable agriculture. According to Niguyen et al. (2017), dietary supplementation of the cocktail protease and xylanase enzyme component improved broiler performance and excreta quality, indicating potential benefits for poultry production efficiency and environmental sustainability. In another study, Lei et al. (2018) concluded that dietary supplementation of xylanase in corn–soybean–meal–wheat-based diets increased egg quality parameters and excreta lactic acid bacteria count, while showing no significant effects on production performance or nutrient digestibility in laying hens. Nutritionists have increasingly recognized the importance of both microbiota and host, with the gut microbiota’s relationship to animal health being influenced by nutrition and the environment (Khan and Naz 2013; Khan et al. 2014). The initial weeks post-hatch play a crucial role in shaping gut-associated immunity, dependent on microflora acquired through early-life feeding and environment (Shah et al. 2020). While the poultry immune system is only partially developed at hatch (Schat and Myers 1993), and the gastrointestinal tract is initially sterile (Harrow et al. 2007), it gradually matures by the age of 3 weeks. Therefore, providing an optimal environment and feed is essential for the optimal development of both systems (Imtiaz et al. 2023; Subhan et al. 2023).

The practice of early probiotic feeding in poultry originated in the early 1970s with the influential study by Nurmi and Rantala (1973) demonstrating successful elimination of *Salmonella enteritidis* in newly hatched chicks through the administration of gut contents obtained from healthy adult chickens. This pioneering approach aimed to establish a healthy microflora in the chicks, leading to competitive exclusion of harmful bacteria (Gul and Alsayegh 2022; Landy and Kavyani 2013). Probiotic as a feed additive has proven effective in promoting the development of a beneficial microflora (Rashid et al. 2022), a critical factor in maintaining intestinal health , reducing the colonization of pathogenic microbes. Consequently, this improvement in chicken productivity has been linked to enhanced broiler performance (Landy and Kavyani 2013; Nusairat et al. 2022; Bidura et al. 2023).

The primary challenge in incorporating feed ingredients into poultry diets lies in the high fiber content, particularly non-starch polysaccharides (NSPs), which resist digestion in poultry due to the absence of necessary enzymes. NSPs, found notably in common energy sources like corn and wheat, lead to nutrient entrapment and increased digesta viscosity, limiting maximum energy digestibility in poultry diets. Studies indicated that approximately 450 kcal of energy of feed remains unused due to NSPs. Exogenous enzymes, such as xylanase, prove effective in releasing energy from fiber fractions, enhancing broiler performance by reducing nutrients available for microorganisms in the lower gut. Furthermore, the incorporation of xylanases into monogastric animal diets provides an additional benefit: xylanase breaks down NSPs into prebiotic compounds, such as xylose and arabinose. These compounds act as prebiotics, fostering the growth of beneficial bacteria in the lower gut, as demonstrated by Ding et al. (2018), who found that xylooligosaccharides stimulate the growth of *Lactobacilli* and *Bifidobacteria*. Consequently, xylanase not only improves digestibility but also enhances gut health by supplying prebiotics for the nourishment of beneficial bacteria (Van Hoeck et al. 2021).

It is inferred that combining probiotic and xylanase could yield positive effects on both the host’s nutrition and microorganisms, fostering a symbiotic relationship. This relationship involves the simultaneous provision of a probiotic and fiber-degrading enzyme to both the beneficial microorganisms and the host ultimately contributing to a balanced and healthy microbiota. Consequently, this study aimed to examine the impact of the combined effects of xylanase and probiotics on live performance, immune response, microbial load, and digestibility in Japanese quails.

## Materials and methods

### Experimental design and diets

A total of 2000 one-day-old Japanese quails were acquired from a commercial hatchery and distributed randomly into floor pens measuring 305 cm × 137 cm. Each treatment consisted of 10 replicate pens, with each pen containing 50 chicks, and the quails were raised until 35 days of age on deep litter. The diets were administered with or without a *Bacillus*-based probiotic at a concentration of 7.5 × 10^7^ cfu/kg of feed, derived from environmental strains of *Bacillus* (Danisco Animal Nutrition, Marlborough, UK). Additionally, the diets included an enzyme, *Trichoderma reesei* endo-xylanase (EnzaPro, BioResource International Inc., Durham, NC, USA), at a concentration of 2,000 U/kg of feed, either alone or in combination. This resulted in four experimental groups, including a control group, a second group fed with probiotics, a third group with enzyme supplementation, and a fourth group receiving both enzyme and probiotic supplementation. The diets consisted of mash starter and finisher formulations according to National Research Council (1994). The composition of the basal diet for quails, presented in Table 1, remained consistent across all groups, and uniform management practices were applied, with the addition of drinkers and feeders to each birdcage.

**Table 1.** Feed composition of Japanese quails.

Percent constituents (%)	Starter to grower phase (0–21 days)	Finisher phase (22–35 days)
Maize (yellow)	57	59
Soybean meal (48%)	38	28
Limestone	1.5	1.6
Dicalcium phosphate	1.6	1.7
Salt (NaCl)	0.3	0.3
dl-Methionine	0.16	0.11
Vitamins and minerals premixes^a^	0.50	0.50
Chemical analysis		
Crude protein (%)	22.0	21.0
Metabolizable energy (kcal/kg)	2800	2900
Crude fiber (%)	3.0	3.5
Lysine (%)	1.3	1.3
Calcium (%)	0.95	0.85
Methionine + cysteine (%)	0.82	0.72
Threonine (%)	0.9	0.6
Available phosphorus (%)	0.45	0.45
Sodium (%)	0.2	0.16
Chloride (%)	0.2	0.16

^a^ Provided per kg of diet: vitamin A, 15000 IU; vitamin; vitamin E,15 IU; D3,2500 IU; thiamine, 2 mg; vitamin K3, 2.5 mg; pyridoxine, 5 mg; riboflavin, 5.5 mg; folic acid, 0.7 mg; vitamin B12, 0.12 mg; pantothenic acid, 15 mg; nicotinic acid, 45 mg; biotin, 0.25 mg; iron, 95 mg; copper, 10 mg; zinc, 100 mg; manganese, 100 mg; iodine, 0.5 mg; selenium, 0.3 mg.

### Performance traits

The birds and feed were weighed individually at weekly intervals for live performance measurements. Mortality was recorded as it occurred. These measurements were utilized to determine body weight, feed intake, and feed conversion ratio (FCR), with adjustments made for mortality. At the conclusion of the feeding trial, five quails from each replication was slaughtered to assess dressing yield. The warm weight of the dressed chicken was measured after slaughter and evisceration. The dressing percentage, calculated as the ratio of the dressed weight to the live weight of the birds, was then computed and recorded (Landy et al. 2021).

### Antigen–antibody titer evaluation

Antibody titers for Infectious Bronchitis (IB), Newcastle disease (ND), and Infectious Bursal Disease (IBD) were assessed using the Hemagglutination Inhibition (HI) test (Hafeez et al. 2024). On day 35, blood samples were collected, and serum was isolated through centrifugation. The HI test, conducted in a microtitration plate, involved twofold dilution of serum samples and the addition of normal saline, antigen, and chicken red blood cells (RBCs) to each well. After incubation, antigen and antibody titers were confirmed, providing insights into the immune response against the specified diseases.

### Assessment of parameters for nutrient digestibility

To assess nutrient digestibility, four birds per replicate were transferred to individual experimental cages to collect fecal samples on day 35 of the trial. The gathered samples were then analyzed for dry matter (DM), ash, crude protein (CP), crude fiber (CF), and ether extract (EE) using standard procedures.

### Digestibility of apparent metabolizable energy

To quantify the apparent metabolizable energy (AME), a bomb calorimeter (BC) standardized with benzoic acid was employed. For the AME calculation, the pelleted sample was incinerated in the bomb calorimeter. The formula below was utilized to estimate the apparent metabolizable energy on a dry matter basis:

$$\begin{array}{c} \text{AME }=\;\left(\text{Energy consumption }-\text{ Energy lost}\right)/\\ \text{ Feed consumption} \end{array}$$

### Cecal microbiota

Cecal digesta samples were gathered from 10 birds in each dietary group using a sterile collection tube and subsequently stored at −20 °C for microbiota colonization assessment (Alharthi et al. 2023). The colonies, ranging from 50 to 300, were visibly distinct and easily countable. A total of 10 μL of the samples were cultured on specific media corresponding to the bacterial species under investigation. *Lactobacillus* spp. were cultured on MRS agar (Hi Media, Mumbai, India), while *Salmonella* spp. and Escherichia coli were cultured on EMB (Hardy Diagnostics, Santa Maria, CA, USA). Colony counting was performed using a colony counter, and the results were expressed in log10 colony-forming units per gram (log10 CFU/1 g digesta).

### Statistical analysis

Statistical analysis of data for all parameters was conducted utilizing SAS software (SAS Institute 2004), employing a one-way analysis of variance with a completely randomized design. The differentiation of means was established through Tukey’s honest significant difference test.

## Results

### Growth performance

The impact of probiotic and enzyme, either individually or in combination, on the feed intake of Japanese quail is presented in Table 2. The results revealed no significant difference in feed intake between the control and experimental groups.

**Table 2.** Effect of probiotic and enzyme individually or in combination on feed intake of Japanese quail.

Group	Weekly feed intake of quail (g)				Cumulative feed intake
	Week 2	Week 3	Week 4	Week 5	
Probiotic	79.86 ± 0.56	102.23 ± 2.0	130.10 ± 1.0	155.13 ± 1.05	468.30 ± 4.07
Enzyme	80.10 ± 2.0	100.20 ± 2.0	129.77 ± 2.08	155.10 ± 2.0	465.17 ± 3.7
Probiotic + enzyme	79.100 ± 1.0	101.10 ± 2.0	129.17 ± 2.07	154.20 ± 2.64	463.63 ± 2.6
Control	80.46 ± 1.51	100.20 ± 2.0	129.30 ± 1.95	154.20 ± 2.0	464.23 ± 4.9
*p* value	0.45	0.585	0.843	0.741	0.101

Mean values in each group represent 500 quails.

The effect of probiotic and enzyme, either individually or in combination, on the weight gain of Japanese quail is given in Table 3. The findings indicated that weekly and overall weight gain was significantly higher (*p* < 0.05) in quails supplemented with both probiotic and enzyme. In week 2, a significantly higher weight gain (*p* < 0.04) was observed in quails treated with probiotic + xylanase compared to both the control group and the group treated with probiotics alone. There was no significant difference in weight gain during week 2 between the control group and the group supplemented with probiotics alone. During weeks 3 and 5, significantly higher weight gain (*p* < 0.02) was observed in quails supplemented with probiotic + xylanase compared to both the control group and the other treatments. However, there was no significant difference in weight gain among the control group, the probiotic group, and the xylanase treatment group. In week 4, a similar trend was observed, except no significant difference was found in weight gain between the quails supplemented with probiotic + xylanase and those supplemented with probiotics alone. The effect of probiotic and enzyme, either individually or in combination, on the FCR of Japanese quail is depicted in Table 4. The results demonstrated that the mean weekly and overall FCR were significantly lower (*p* < 0.05) in quails supplemented with both probiotic and enzyme compared to the control. During week 2, a notable improvement in FCR was noted in quails supplemented with probiotic + xylanase compared to those in the probiotic and control groups. However, there was no significant difference in FCR between the probiotic + xylanase group and quails supplemented solely with xylanase. In week 3, a similar trend was observed, but FCR was significantly (*p* < 0.01) improved only in quails supplemented with probiotic + xylanase. Likewise, in week 4, a significantly lower FCR (*p* = 0.03) was observed in quails supplemented with probiotic + xylanase compared to both the control group and quails supplemented solely with xylanase, with no significant difference observed compared to those supplemented with probiotics alone. A similar trend was observed in week 5, although no significant difference was noted between the xylanase and probiotic + xylanase supplemented groups.

**Table 3.** Effect of probiotic and enzyme individually or in combination on weight gain of Japanese quail.

Group	Weekly body weight gain of quails (g)				Cumulative body weight
	Week-2	Week-3	Week-4	Week-5	
Probiotic	34.16^b^ ± 2.0	45.26^b^ ± 3.0	52.43^ab^ ± 2.0	52.16^b^ ± 1.0	183.57^b^ ± 1.6
Enzyme	36.20^ab^ ± 1.0	47.30^b^ ± 3.0	50.30^b^ ± 1.0	54.50^b^ ± 2.6	188^b^ ± 1.8
Probiotic + enzyme	39.90^a^ ± 2.0	53.10^a^ ± 2.0	54.50^a^ ± 1.5	58.30^a^ ± 2.0	206.47^a^ ± 1.4
Control	35.16^b^ ± 2.6	47.53^b^ ± 1.5	49.83^b^ ± 1.5	51.43^b^ ± 1.5	183.87^b^ ± 2.6
*p* value	0.04	0.02	0.02	0.008	0.004

Means in similar column with dissimilar superscripted are significantly differ at (*p* < 0.05).

Mean values in each group represent 500 quails.

**Table 4.** Effect of probiotic and enzyme individually or in combination on feed conversion ratio of Japanese quail.

Group	Weekly feed conversion ratio (FCR)				Cumulative FCR
	Week-2	Week-3	Week-4	Week-5	
Probiotic	2.34^a^ ± 0.15	2.26^a^ ± 0.14	2.47^ab^ ± 0.1	2.99^a^ ± 0.08	2.43^a^ ± 0.12
Enzyme	2.21^ab^ ± 0.0	2.12^a^ ± 0.12	2.56^a^ ± 0.05	2.85^ab^ ± 0.16	2.47^a^ ± 0.08
Probiotic + enzyme	1.98^b^ ± 0.07	1.90^b^ ± 0.06	2.36^b^ ± 0.1	2.64^b^ ± 0.04	2.24^b^ ± 0.01
Control	2.26^a^ ± 0.19	2.10^a^ ± 0.02	2.59^a^ ± 0.04	2.99^a^ ± 0.12	2.52^a^ ± 0.07
*p* value	0.04	0.01	0.03	0.01	0.01

Means with dissimilar superscripts in similar column significantly different at (*p* < 0.05).

Mean values in each group represent 500 quails.

### Carcass quality and mortality

The effect of probiotic and enzyme, either individually or in combination, on the dressing percentage and mortality of Japanese quail is shown in Table 5. The mean dressing percentage was significantly higher (*p* < 0.05) in the experimental groups compared to the control, while mortality was significantly lower in the birds supplemented with a combination of enzyme and probiotic. Dressing and mortality percentage did not vary significantly (*p* = 0.04) between xylanase and probiotic + xylanase supplemented groups.

**Table 5.** Effect of probiotic and enzyme individually or in combination on dressing percentage and mortality of Japanese quail.

	Dressing percentage	Mortality (%)
Probiotic	55.57^ab^ ± 3.54	1.33^a^ ± 0.57
Enzyme	58.68^a^ ± 2.2	1.0^ab^ ± 0.0
Probiotic + enzyme	59.08^a^ ± 3.07	0.33^b^ ± 0.57
Control	52.07^b^ ± 1.62	1.66^a^ ± 0.57
*p* value	0.04	0.05

Means with dissimilar superscripts in similar column differ significantly at (*p* < 0.05).

Mean values in each group for dressing percentage and mortality represent 50 and 500 quails, respectively.

### Immune response

The impact of probiotic and enzyme, either individually or in combination, on the antibody titre of Japanese quail is presented in Table 6. The results showed that the mean antibody titre against IB and IBD was significantly higher (*p* < 0.05) in quails receiving combined probiotic and enzyme supplementation. However, the antibody titre against ND virus was significantly higher in the groups supplemented with probiotic and enzyme, either individually or in combination, compared to the control. Furthermore, the antibody titers for IB and ND did not show significant variation (*p* = 0.04) among groups supplemented with probiotic, xylanase, and probiotic + xylanase. However, the antibody titer for IBD did not significantly differ (*p* = 0.02) between the control group and the groups supplemented with xylanase or probiotic + xylanase.

**Table 6.** Effect of probiotic and enzyme individually or in combination on antibody titre against different infectious diseases of Japanese quail.

Groups	Antibody titer against vaccination		
	Infectious bronchitis	Infectious bursal disease	New castle disease
Probiotic	4.41^ab^ ± 0.57	4.42^b^ ± 0.19	4.74^a^ ± 0.0.3
Enzyme	4.66^ab^ ± 0.01	4.52^b^ ± 0.04	4.88^a^ ± 0.55
Probiotic + enzyme	4.92^a^ ± 0.01	5.03^a^ ± 0.48	4.99^a^ ± 0.47
Control	4.12^b^ ± 0.01	4.22^b^ ± 0.09	4.04^b^ ± 0.01
*p* value	0.04	0.02	0.04

Means with dissimilar superscripts in similar column differ significantly at (*p* < 0.05).

Mean values in each group represent 50 quails.

### Nutrients digestibility

The effect of probiotic and enzyme, either individually or in combination, on the digestibility of DM, CP, EE, CF, NFE, and ash in Japanese quail is shown in Table 7. The results revealed significantly higher (*p* < 0.05) digestibility in the groups receiving combined enzyme and probiotic supplementation compared to the control. No significant difference was found in the concentration of DM, EE, CF, NFE, and ash between the enzyme group and the combination of enzyme and probiotic. Additionally, no significant difference was observed in nutrient digestibility between the control and the probiotic-supplemented quails. The digestibility of DM showed no significant variation (*p* = 0.01) between groups supplemented with xylanase and those supplemented with probiotic + xylanase. Similarly, the digestibility of CP, EE, and CF remained statistically unchanged across groups supplemented with probiotic, xylanase, and probiotic + xylanase. Additionally, NFE and ash did not exhibit significant differences (*p* = 0.02) between quails supplemented with probiotic and those supplemented with xylanase. The observed improvements in digestibility with combined enzyme and probiotic supplementation likely stem from synergistic effects on gut health and nutrient metabolism. While individual supplementation did not significantly alter nutrient concentration or digestibility, the combined approach may optimize nutrient utilization pathways, leading to enhanced overall digestibility in quails. The results showed that AME was significantly higher (*p* < 0.05) in the groups receiving enzyme and the combination of enzyme and probiotic supplementation compared to the control. No significant (*p* = 0.01) difference was observed xylanase and probiotic and xylanase supplemented quail for AME. Similarly, the control and the probiotic groups did not show any significant change in AME.

**Table 7.** Effect of probiotic and enzyme individually or in combination on nutrients digestibility of Japanese quail.

Group	Digestibility coefficient (%)						
	Dry matter	Crude protein	Ether extract	Crude fat	Nitrogen-free extract	Ash	Apparent metabolizable energy (AME) (MJ/kg)
Probiotic	66.35^bc^±0.99	66.23^b^ ± 1.01	64.36^b^ ± 1.01	61.88^b^ ± 1.14	41.58^bc^ ± 1.50	21.50^bc^ ± 1.01	12.14^b^ ± 1.21
Enzyme	67.91^ab^ ± 1.50	67.09^b^ ± 1.52	65.31^ab^ ± 1.02	62.80^ab^ ± 0.59	42.88^ab^ ± 1.51	22.47^ab^ ± 1.02	12.17^a^ ± 1.45
Probiotic + enzyme	69.44^a^ ± 1.03	69.53^a^ ± 1.00	66.47^a^ ± 1.01	64.41^a^ ± 1.54	44.22^a^ ± 1.01	23.54^a^ ± 0.97	12.18^a^ ± 1.66
Control	65.13^c^ ± 1.53	65.62^b^ ± 0.96	63.51^b^ ± 1.02	60.98^b^ ± 1.51	40.31^c^ ± 0.92	20.39^c^ ± 0.99	12.13^b^ ± 1.82
P- value	0.01	0.01	0.03	0.05	0.02	0.02	0.01

Means with different superscripts in column differ significantly at (*p* < 0.05).

Mean values in each group represent 40 quails per treatment.

### Cecal microbiota

Table 8 outlines the effect of probiotic and enzyme, either individually or in combination, on cecal microbiota and apparent metabolizable energy (AME) of Japanese quail. Furthermore, the population of *LactoBacillus* increased significantly (*p* < 0.05) in the groups receiving probiotic, enzyme, and the combination of probiotic and enzyme supplementation. However, *Lactobacillus* did not vary among probiotic, xylanase and combination of probiotic and xylanase supplemented quails. Additionally, the populations of *E. coli* and *Salmonella* in the ceca decreased significantly (*p* < 0.05) in quails supplemented with both probiotic and enzyme. However, *E. coli* and *Salmonella* count did not vary among probiotic, xylanse and combination of probiotic and xylanase supplemented quails.

**Table 8.** Effect of probiotic and enzyme individually or in combination on cecal microbiota of Japanese quail.

Group	Gut microbiota (log_10_ cfu/g)		
	*Lactobacillus*	*E. coli*	*Salmonella*
Probiotic	2.33^ab^ ± 0.57	3.33^ab^ ± 0.57	3.00^b^ ± 0.00
Enzyme	2.66^ab^ ± 0.57	3.66^ab^ ± 0.57	3.33^ab^ ± 0.57
Probiotic + enzyme	3.66^a^ ± 1.15	2.66^b^ ± 0.57	2.66^b^ ± 0.57
Control	1.33^b^ ± 0.57	4.00^a^ ± 0.00	4.00^a^ ± 0.00
*p* value	0.03	0.05	0.02

Means with different superscripts within a column differ significantly at (*p* < 0.05).

Mean values in each group represent 40 quails per treatment.

## Discussion

In the present study, dietary supplementation did not influence feed intake; however, a significant improvement in weight gain, feed conversion ratio (FCR) and dressing percentage was observed in response to the combined supplementation of probiotics and enzymes. In this study, growing quails were given a diet supplemented with either xylanase alone, probiotic alone or a combination of both the enzyme and probiotic. Recognizing the potential complementary modes of action of exogenous digestive enzymes and probiotics, the concurrent use of these two products may offer more benefits compared to their individual application.

According to Yeo and Kim (1997), this enhanced growth performance could be attributed to the establishment of a well-balanced microflora facilitated by the probiotic present in the diet. The absence of differences in feed intake aligns with previous studies that assessed the individual inclusion of enzymes and probiotics (Nayebpor et al. 2007; Cowieson and Ravindran 2008; Meng et al. 2010; Waititu et al. 2014; Nusairat et al. 2022). Despite the concurrent use of enzymes and probiotics in the current trial, no variations were noted in feed consumption, consistent with findings in prior research (Flores et al. 2016). Nayebpor et al. (2007) reported an increase in body weight at days 21, 28, and 42 compared to the control in broilers. While there are reports of enhanced broiler performance with probiotic supplementation, conflicting results exist, with instances where probiotic supplementation had no effect on body weight. These inconsistencies may be attributed to other factors influencing the efficacy of the probiotic. Singh et al. (2021) found that supplementing with xylanase enhanced the growth of beneficial gut bacteria in broilers, leading to improved overall performance. This observation supports the idea that there is a synergistic effect when combining probiotics and xylanase. Chen et al. (2018) proposed that the incorporation of xylanase could notably enhance broiler carcass characteristics. The increased carcass weights observed in chickens fed xylanase or probiotic diets can be partially attributed to the improved utilization of nutrients, particularly amino acids. Murugesan and Persia (2013) and Murugesan et al. (2014) demonstrated that broilers fed corn- and soybean meal-based diets exhibited a higher feed conversion ratio (FCR) when provided with a combination of probiotics and enzymes compared to when the additives were used individually. Additionally, Momtazan et al. (2011) identified a linear interaction in growth performance, indicating a correlation between the concentration of the enzyme complex and the inclusion of probiotics. The study demonstrates that combined enzyme and probiotic supplementation significantly improves nutrient digestibility in quails compared to control groups. While individual enzyme or probiotic supplementation did not yield significant improvements, the synergistic effect of combining both supplements suggests a promising strategy for enhancing nutrient utilization in poultry production.

In the currents study, probiotic and xylanase supplementation improved nutrients digestibility and AME in comparison to the control. The response of digestability of several nutreints such as DM, EE, CF, NFE and AME in response to xylanase was also encouraging. Xylanase primarily works by decreasing the viscosity of digesta, a phenomenon more pronounced when the dietary fiber, particularly non-starch polysaccharides (NSPs), is elevated. The soluble fraction of NSPs, primarily arabinoxylans, hinders nutrient digestion and absorption by encapsulating nutrients. This interference reduces the effectiveness of endogenous enzymes in nutrient hydrolysis. Additionally, high-fiber diets lead to increased mucous production, further hindering nutrient transport through epithelial cells. Xylanase’s capacity to break down glycosidic bonds in NSPs helps reduce digesta viscosity, releasing nutrients as free sugars and mitigating the overall anti-nutritional effects of NSPs (Nusairat et al. 2022). Inclusion of soybean hulls in diets, with heightened fiber content and reduced energy, poses a challenging environment for xylanase to showcase its potential. This scenario serves as an exemplary illustration of the challenges faced by the contemporary poultry industry. The addition of xylanase to monogastric diets lowers digesta viscosity and enhances nutrient utilization by breaking down non-starch polysaccharides (NSPs) into mono and oligosaccharides. This improvement is evident in the enhanced digestibility of energy observed in this trial. Additionally, researchers noted that the probiotic effects of B. subtilis were more prominent in broiler diets deficient in nutrients, leading to an enhanced energy digestibility (Goodarzi Boroojeni et al. 2018). The improved growth performance can be attributed to the probiotic’s ability to produce lactic acid, which reduces the pH of the intestinal content. This acidic environment inhibits the growth of invasive pathogens, contributing to enhanced nutrient digestibility. By improving the efficiency of digestion and nutrient absorption processes, the probiotic ultimately leads to increased growth performance in this study (Ngyuyen and Kim 2020). The variations in digestibility among supplementation groups suggest that the combined enzyme and probiotic supplementation synergistically enhanced nutrient absorption, leading to significantly higher digestibility compared to the control. Despite no significant differences in nutrient concentration or digestibility between enzyme and combination groups, the combined approach likely optimized nutrient utilization pathways, resulting in improved overall digestibility in quails.

In the current study, combination of probiotic and xylanase was superior in comparison to the individual effect of xylanse and the probiotic. Studies have demonstrated that probiotics can enhance the immune response against antigens (Teo and Tan 2007). Birds fed diets with a combination of probiotics and enzymes exhibited a satisfactory immune response compared to a control group, as reported by Seidavi et al. (2017). The gut microbiome, consisting of diverse bacteria, yeast, and protozoa, is a complex system. Including probiotics in the diet is crucial for maintaining the balance of the intestinal biota. Commensal microorganisms in the intestinal tract are essential for poultry digestion and immunity, as highlighted by Seidavi et al. (2017). The link between robust growth and immunity is rooted in intestinal health. Dietary supplementation, including enzymes and probiotics, seems to contribute to the enhancement of the immune system and growth performance in broilers by fostering intestinal health. The synergistic effects of probiotics and enzymes likely contribute to improved gut health, nutrient utilization, and immune function. However, variations in antibody titers among different supplementation groups suggest specific effects on immunity against different pathogens. Further research into the optimal supplementation strategies and mechanisms underlying these effects is warranted.

Nusairat et al. (2018) reported the positive impact of a combination of xylanase and probiotics in reducing *Salmonella* incidence in broilers at 21 and 42 days. Various studies have explored the efficacy of different DFM with gut-health-enhancing properties in both chickens and turkeys. Supplementation with *Lactobacilli* was found to decrease cecal coliform counts in both turkeys and broilers (Francis et al. 1978; Watkins and Kratzer 1983), as well as to reduce *Salmonella* enteritidis and Clostridium perfringens in chickens (Kizerwetter-Swida and Binek 2009). Additionally, xylanase supplementation alone has demonstrated the ability to decrease *E. coli* counts and increase *Lactobacillus* spp. counts in the ceca of 35-day-old broilers (Van Hoeck et al. 2021). Therefore, the observed reduction in microbial load could be attributed to the combined effect of both multi-strain probiotic and xylanase. The decrease in pathogen counts in the ceca suggests less contamination of the environment. This, in turn, helps control the spread of infection within the flock and subsequently to other flocks. The addition of xylanase has the potential to enhance the digestion of non-starch polysaccharides, thereby providing the necessary substrates for the proliferation of probiotic bacteria. Xylanase can facilitate the breakdown of starch, a process that might potentially restrict the availability of substrates for pathogenic bacteria. If the mechanisms of enzymes and probiotics can reach their full potential, it logically follows that the combination of these products may contribute to enhancing intestinal health and achieving a balanced microbial environment in the gut. This, in turn, could lead to improved growth performance and feed efficiency in broilers (Flores et al. 2016). Dersjant-Li et al. (2015) reported that the combination of multi-enzymes with direct-fed microbials (DFM) could positively influence microbial balance and the production of microbial metabolic end-products in the small intestine. This combination stimulates the growth of beneficial bacteria, such as Lactobacilli, while reducing the colonization of pathogenic bacteria like *Clostridium perfringens*. The observed increase in total short-chain fatty acid and lactic acid production suggests improved intestinal health as a potential outcome of this synergistic approach. The differential changes in *Lactobacillus* populations could be due to the specific strains used in the probiotic supplementation and their interactions with gut microbiota, while the decrease in *E. coli* and *Salmonella* counts might result from the combined antimicrobial properties of both probiotic and enzyme supplementations.

## Conclusion

From the results of the present study, it can be concluded that the supplementation of xylanase enzyme and probiotic in Japanese quails led to enhanced growth performance, improved nutrient digestibility, enhanced immune response, reduced cecal *E. coli* and *Salmonella* and improved *Lactobacillus* population.

## Data Availability

This data is available is available in student thesis.
